# Parametric excitation of multiple resonant radiations from localized wavepackets

**DOI:** 10.1038/srep09433

**Published:** 2015-03-24

**Authors:** Matteo Conforti, Stefano Trillo, Arnaud Mussot, Alexandre Kudlinski

**Affiliations:** 1 PhLAM/IRCICA, CNRS-Université Lille 1, UMR 8523/USR 3380, F-59655 Villeneuve d'Ascq, France; 2 Dipartimento di Ingegneria, Università di Ferrara, Via Saragat 1, 44122 Ferrara, Italy

## Abstract

Fundamental physical phenomena such as laser-induced ionization, driven quantum tunneling, Faraday waves, Bogoliubov quasiparticle excitations, and the control of new states of matter rely on time-periodic driving of the system. A remarkable property of such driving is that it can induce the localized (bound) states to resonantly couple to the continuum. Therefore experiments that allow for enlightening and controlling the mechanisms underlying such coupling are of paramount importance. We implement such an experiment in a special optical fiber characterized by a dispersion oscillating along the propagation coordinate, which mimics “time”. The quasi-momentum associated with such periodic perturbation is responsible for the efficient coupling of energy from the localized wave-packets (solitons in anomalous dispersion and shock fronts in normal dispersion) sustained by the fiber nonlinearity, into free-running linear dispersive waves (continuum) at multiple resonant frequencies. Remarkably, the observed resonances can be explained by means of a unified approach, regardless of the fact that the localized state is a soliton-like pulse or a shock front.

Our experimental set-up realizes a platform for studying the coupling of localized states of a nearly conservative (Hamiltonian) system into radiation modes induced by periodic driving. A sketch of the physical principle is depicted in Fig. 1. Time periodic driving of the system rules fundamental physical phenomena such as laser-induced ionization^1^, driven quantum tunneling^2^, Faraday waves^3^,^4^,^5^,^6^, Bogoliubov quasiparticle excitations^7^, and the control of new states of matter^8^,^9^,^10^,^11^. The localized states that we exploit are nonlinear (non-spreading) wavepackets of optical fibers, namely temporal solitons^12^,^13^ and dispersive shock waves (DSWs)^14^,^15^,^16^. They share the property to have a well-defined wavenumber and group-velocity which are crucial for determining their resonances. Their excitation is readily accessible by operating with opposite group-velocity dispersion (GVD, *β*_2_ = *d*^2^*k*/*dω*^2^): anomalous (*β*_2_ < 0) for solitons in order to balance nonlinearity, whereas DSWs emerge from a gradient catastrophe occurring in the normal GVD (*β*_2_ > 0). Despite such difference, both are essentially described by a Hamiltonian model, namely the nonlinear Schrödinger equation (NLSE)^12^,^13^, where the propagation distance plays the role of evolution variable (usually time). This allows us to introduce periodic driving by employing photonic crystal fibers whose flexibility to engineer dispersion is fully exploited to have a longitudinal periodic GVD, thus realizing dispersion oscillating fibers (DOFs)^17^. We report clear evidence that this built-in and tailorable periodicity of the dispersion is responsible for the parametric excitation of radiation modes which are amplified out of quantum fluctuations at multiple resonant frequencies (even for a spatially harmonic variation). This thus constitutes a novel implementation of quasi-phase-matching^18^. This is in marked contrast with radiation caused by standard perturbations such as third-order (or higher) dispersion^19^,^20^,^21^,^22^,^23^,^24^, which usually feature isolated resonances. Our results also clearly show that the parametric excitation of resonances is neither a prerogative of solitons nor of systems with periodic injection or extraction of energy such as fiber lasers^25^, passive cavities^26^ or lumped amplifier links^27^,^28^ where dissipative resonances lead to the generation of so-called Kelly sidebands.

## Results

### Theory

Perturbation theory allows us to predict the frequency detunings *ω_RR_* of the resonant radiations (RR) that can be parametrically excited in a DOF. They turn out to be given by the roots of the following expression (see Supplementary information for a detailed derivation):
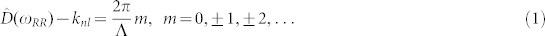
where 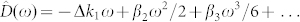 is the average linear dispersion in the pump reference frame. *β*_3_ ≡ *d*^3^*k*/*dω*^3^ and Δ*k*_1_ arises from the deviation of the actual group-velocity from the natural one^22^). In Eq. (1), *k_nl_* is a well defined nonlinear wavenumber fixed by power. For a bright soliton *k_nl_* = *γP*/2, where *P* and *γ* are the soliton peak power and the fiber nonlinearity, whereas for a shock wave *k_nl_* = −*γP_b_*, being *P_b_* the power of the background field over which the RR propagates^23^. Equation (1) generalizes previously proposed formulas^31^,^32^ and expresses momentum conservation: it states that the difference between the momentum of the linear waves and the momentum generated by the nonlinear pump must be equal to the virtual momentum carried by the periodic modulation of the dispersion.

**Figure 2:**
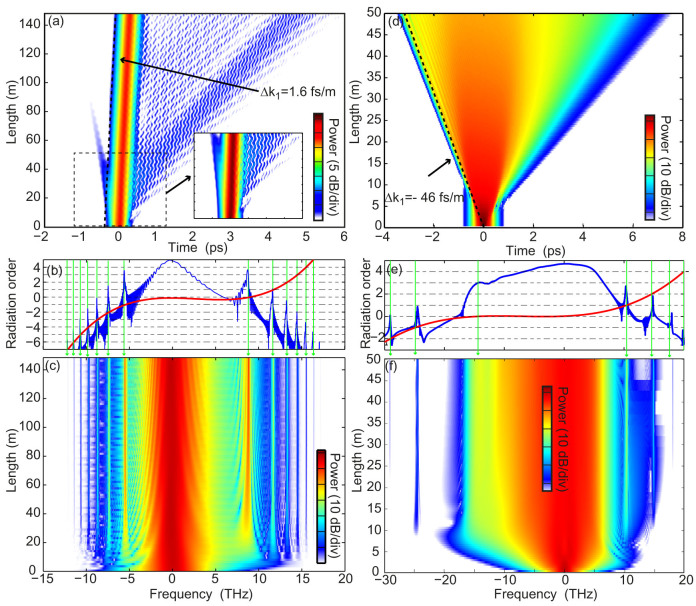
Parametrically excited multiple resonances at fixed power (numerical modeling). Left panels a,b,c refer to the soliton pumped configuration, whereas right panels d,e,f refer to the shock configuration. (a,d) colormap of the temporal evolution of power (log scale) along the fiber; (b,e) output spectra superimposed on the dispersion relation 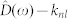 (red curve). The vertical green lines correspond to the resonances determined by the graphical solution of Eq. (1), i.e. the crossing between the dispersion curve and the dashed horizontal lines that corresponds to integer multiples of quasi-momentum 2*π*/Λ; (c,f) colormap evolution of the spectrum along the fiber. All the results are obtained by numerical simulation of the NLSE (see methods), with periodic GVD and *β*_3_ as additional parameter.

We verified that Eq. (1) describes accurately the parametric excitation of RR in both GVD regimes. A clear illustration of this phenomenon is provided in Fig. 2, which shows the simulated evolution of a hyperbolic secant pulse (150 fs duration, peak power *P*) based on the NLSE with included periodic GVD and dispersion slope *β*_3_ (see methods). Figure 2a displays the time domain evolution of a pulse with *P* = 15 W, corresponding to a nearly fundamental soliton launched in a 150 m long DOF of period Λ = 5 m. Strong radiation traveling slower than the soliton becomes visible beyond the activation distance *z_a_* ~ 20 m, corresponding to maximum pulse compression. In the spectral domain, the radiation modes correspond to several distinct and well defined frequencies [see Fig. 2b,c] that agree with the prediction based on Eq. (1)(vertical green lines). The peak detuned by ~9 THz from the pump turns out to be the standard RR [*m* = 0 in Eq. (1)], whereas other twelve peaks originate from the periodic perturbation [*m* ≠ 0 in Eq. (1)].

When pumping in the normal GVD regime, we selected a higher peak power and duration (*P* = 100 *W*, 280 *fs*) in order to access wave-breaking, and a shorter modulation period (Λ = 0.5 *m*) in order to have resonances in a realistic frequency span. In this case, the localized state is the shock front that emerges over the pulse leading edge after the breaking and activation distance *z* ~ 8 m, which is clearly visible in Fig. 2d. Also in this regime, we identify a first spectral peak at −15 THz in the anomalous GVD region [see Fig. 2e,f] as the standard RR (*m* = 0 mode) due to *β*_3_, while other five peaks are clearly visible. These are parametrically excited resonant modes that correspond to *m* = ±1, ±2, 3 which position are also well predicted by Eq. (1)(see vertical green lines).

We emphasize that in both configurations, the *m* = 0 isolated resonance lies in the opposite GVD regime with respect to the localized wavepackets that generate it, and disappear for *β*_3_ = 0. Conversely, the parametric resonances with *m* ≠ 0 lie in both normal and anomalous GVD regimes. They also survive the absence of higher-order dispersion (*β_n_* = 0, *n* ≥ 3), and their number is infinite for a harmonic perturbation. In practice, their actual number is limited because their amplitude decreases with the frequency shift and they become weaker than the pulse spectrum envelope and/or the noise background. Note also that it is important to take into account the group-velocity deviation Δ*k*_1_ [see Fig. 2a,d] in the 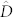 term of Eq. (1) in both configurations, in order to accurately predict the actual values of the resonances.

**Figure 3:**
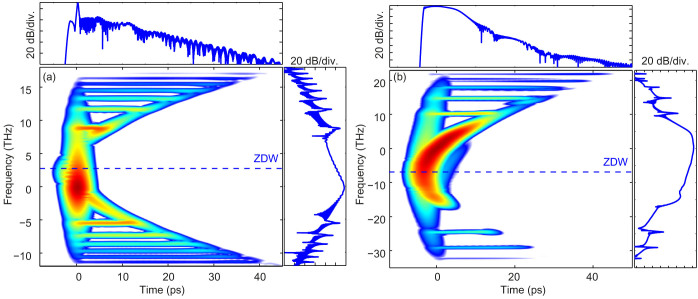
Spectrograms corresponding to evolutions in Fig. 2 (numerical modeling). (a) soliton configuration. (b) shock wave configuration. The spectrograms represent the spectra over gated time intervals and are computed at the output of each fibers (for the evolution along the fibers, see the additional multimedia files). ZDW: zero-dispersion wavelength.

The nature of coupling process is further clarified by the spectrograms corresponding to Fig. 2, which are displayed in Fig. 3. In both cases, the pump wave-packets remain clearly localized in both temporal and spectral domains, while shedding energy into radiation modes, which disperse in time while remaining at the frequencies predicted by the resonances in Eq. (1). We have found that the localized pumps experience temporal and spectral breathing with the period of the perturbation and refill the radiation at each cycle of spectral broadening (see multimedia files). In spite of this transfer of energy the radiation damping of the pump is small, thus confirming the metastable nature of these wave-packets^33^.

### Experiments

**Figure 4:**
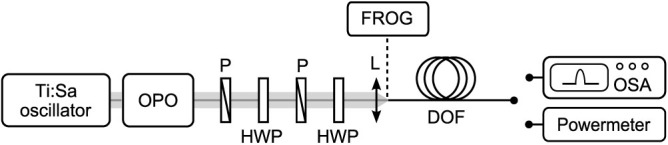
Experimental set-up P: polarizer; HWP: half-wave plate.

**Figure 5:**
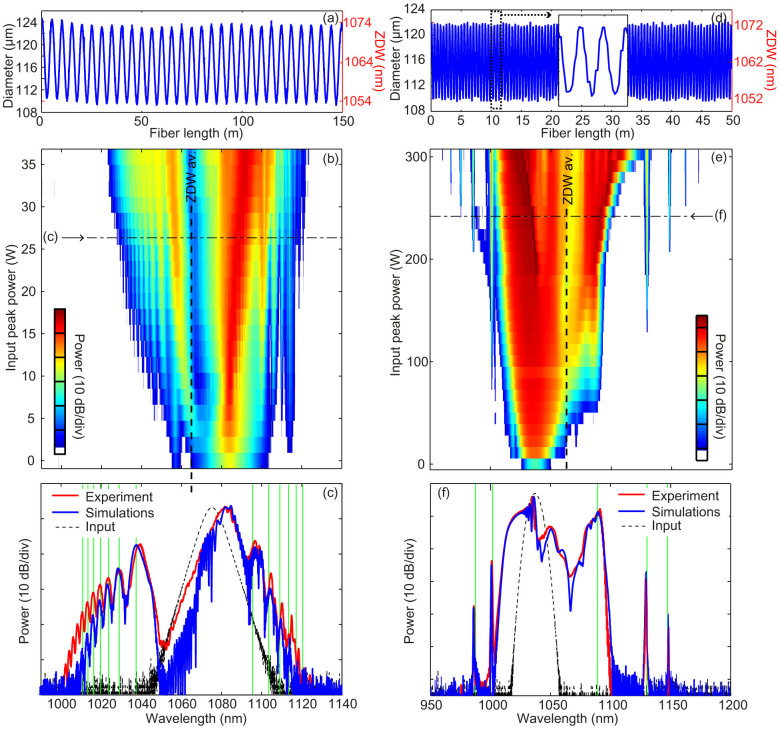
Parametrically excited multiple resonances from a fundamental soliton (left panels) and from a DSW (right panels): experimental results. (a,d): Longitudinal evolution of the DOF diameter (left vertical axis) and consequent zero-dispersion wavelength (ZDW, right vertical axis) used for the soliton (a) and dispersive shock waves (d) experiments. (b,e): Experimental power maps showing the development of asymmetric sidebands with increasing pump peak power. (c,f): Comparison between experimental (red lines) and simulated spectra (blue lines) for a pump peak power of 26.4 W in the soliton case (c) and of 234 W in the dispersive shock wave case (f). Vertical green lines depict the resonance from Eq. (1).

We have designed two experiments in order to observe the parametrically excited resonances from both solitons and DSWs. The experiment, sketched in Fig. 4 and detailed in methods, simply consists of a femtosecond laser pulse launched in DOFs, whose parameters are close to those used in the illustration examples in Figs. 2–3. Due to experimental constraints, only the initial pulses were slightly different. In a first experiment, a 150 fs pulse centered at 1075 nm was launched in a 150 m-long DOF with a modulation period of 5 m. Figure 5a shows the longitudinal profile of the fiber diameter measured during the draw process (left axis) and the corresponding calculated zero dispersion wavelength (ZDW, right axis). At the pump wavelength of 1075 nm, the average dispersion is slightly anomalous, so that the pump pulse is able to excite a near-fundamental soliton for peak powers in the order of a few tens of watts. An experimental power map, representing the output spectrum recorded for increasing pump peak power, is plotted in Fig. 5b. It shows that, for increasing pump peak power, the spectrum rapidly evolves from a hyperbolic secant shape to a much more structured and highly asymmetric one containing more and more sharp spectral resonances. More precisely, these discrete spectral sidebands corresponds to the parametric excitation of the RR that stems from the periodic variation of the second order dispersion. Numerical simulations of the generalized NLSE (see methods and supplementary information), without any free parameter, also reproduce these features with an excellent quantitative agreement. This can be seen in Fig. 5c, where the experimental spectrum obtained for a pump peak power of 25 W (red line) is compared with the simulated one (blue line). In addition, it is worth noting that the theory is quite robust since the observed peaks are accurately predicted by solutions of Eq. (1) (green vertical lines) in these experiments.

The second experiment presented here is devoted to investigating radiating DSWs. The DOF is shorter (50 m) as well as the modulation period (0.5 m, see longitudinal profile in Fig. 5d). It has been pumped with 280 fs pulses. The pump wavelength was tuned to 1037 nm so that it lies in the normal average dispersion region required to generate a shock wave from a few hundreds of watts of peak power (see additional experimental details in supplementary information). Figure 5e shows the experimental power map. Starting at about 50 W, a RR peak is generated across the average zero dispersion wavelength. For increasing pump peak powers, additional discrete spectral sidebands corresponding to parametrically excited RR appear, similarly to the soliton case. The DOF period Λ being 10 times shorter in this case, the spacing between two adjacent peaks is 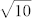 times larger than in the soliton case, as expected from Eq. (1). These results are again in excellent agreement with numerical simulations using a generalized NLSE and with Eq. (1), as shown in Fig. 5f and in supplementary information.

## Conclusions

To summarize, we have demonstrated that localized states (either a soliton or a dispersive shock wave) can efficiently transfer energy to multiple resonant radiations at different frequencies, as a result of the quasi-momentum associated to a DOF. Our experimental results, supported by numerical simulations and by the perturbative analysis that leads to Eq. (1), prove that a DOF is a very simple and highly tailorable platform allowing to study the periodicity-induced coupling of nonlinear bound states to the radiation continuum, which is a general feature in systems driven by a time-periodic Hamiltonian. The DOF platform can also be successfully used to study how this coupling process due to higher order dispersion develop in the presence of a train of random solitons arising from spontanous modulation instability or in driven-damped deformations of Hamiltonian systems such as those describing passive ring resonators.

## Methods

### Simulation

The results illustrated in Figs. 2–3 have been obtained from numerical integration of the following NLSE for the electric field envelope *E* = *E*(*z*,*t*) propagating along the fiber^12^
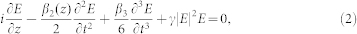


Equation 2 is well known to maintain conservative (Hamiltonian) structure despite the periodic perturbation embedded in the term *β*_2_(*z*) = *β*_2_ + *δβ*_2_sin(2*πz*/Λ) (where *δβ*_2_ is the perturbation amplitude around the average GVD *β*_2_), and the third-order dispersion *β*_3_ (which plays a significant role in experiments performed by pumping close to the ZDW, as in our case). We have employed the following values of the parameters that arise from fiber characterization: *γ* = 10 (W km)^−1^, *δβ*_2_ = 1.2 ps^2^/km, *β*_2_ = −1.2 ps^2^/km and *β*_3_ = 0.0716 ps^3^/km for the soliton configuration, whereas *β*_2_ = −2.9 ps^2^/km and *β*_3_ = 0.0645 ps^3^/km for the DSW configuration. The nonlinear term in Eq. (2) acts as a self-induced potential that allows for the existence of localized states in the form of bright solitons for *β*_2_ < 0 (anomalous dispersion) or dispersive shock waves for *β*_2_ > 0 (normal GVD), whose leading front is, in the unperturbed case, locally a dark soliton^23^. The radiating soliton excited in Fig. 2a,b,c is characterized by a soliton number 
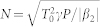
 slightly higher than 1, so that the pulse undergoes temporal compression and hence spectral broadening. It thus acts as an effective seed for the parametric excitation of RR. We emphasize that the periodic perturbation is nearly resonant with the soliton period (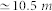), which distinguishes our regime from non-radiating guiding-center or average solitons^29^,^30^, which are normally seen when the periodic perturbation is much faster than the soliton period. In the shock case, the equivalent quantity 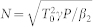 is much larger than unity (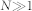) so that the nonlinearity drives the pulse towards the gradient catastrophe that causes the formation of the shock front^15^.

Numerical simulations of Eq. (2) have been performed by using the split step Fourier method with a temporal resolution of 5 fs, 2^14^ points and a spatial resolution ranging from 0.025 m to 0.1 m for the shock and the soliton configurations, respectively.

When directly comparing the numerics with the experimental data (results in Fig. 5), we have accounted also for secondary effects in the fiber such as losses, Raman scattering, self-steepening, and fourth-order dispersion by making use of a generalized NLSE (Eq. (2.3.36) in Ref. 12, also reported explicitly in the Supplemental information). However, we have verified that the impact of such effects on the pulse evolution over the length and temporal scales involved in the experiment is really minor, and most of all does not affect the resonances.

### Experiments

Experiments have been performed exploiting a Ti:Sa oscillator delivering 140 fs full width at half maximum (FWHM) near transform limited pulses. They are sent into an optical parametric oscillator (OPO) allowing to generate tunable slightly chirped femtosecond pulses. The output beam then passes through a combination of two polarizers and two half-wave plates in order to carefully adjust the polarization state and pump power simultaneously. It is launched in the DOF with an aspherical lens. The pulses were characterized with a frequency resolved optical gating (FROG) system, before being injected into the DOF. For the soliton experiment, the OPO was bypassed so that pulses directly coming from the Ti:Sa oscillator were used. They were centred at 1075 nm, and they were measured at 150 fs FWHM at the DOF input (i.e. after the launch lens) with a small chirp *C* = 0.35 (as defined in Ref. 12). For the dispersive shock wave experiment, the OPO was used and tuned to 1037 nm. The pulses at the DOF input were measured at 280 fs FWHM, with a chirp *C* = 1.24. Spectra out of the DOF were acquired with an optical spectrum analyzer with a resolution of 0.2 nm. The output power was measured with a power-meter and the input power was deduced knowing the DOF total attenuation. It was cross-checked by cutting the DOF at the end of the experiment and measuring the power out of the 0.5 m DOF initial section.

## Author Contributions

A.K. carried out experiments. The development of analytical tools was carried out by M.C. and S.T. and simulations were performed by M.C. and A.M. All authors conceived the idea of this work, participated in the analysis and interpretation of the results and in the writing of the paper.

## Supplementary Material

Supplementary Information Supplementary Informations

Supplementary Information Spectrogram (shock case)

Supplementary Information Spectrogram (soliton case)

## Figures and Tables

**Figure 1:**
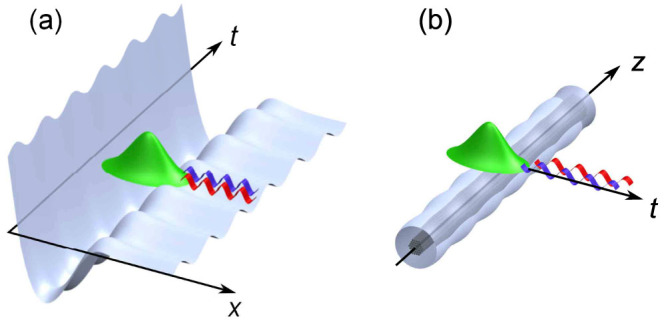
Sketch of physically equivalent phenomena. (a) coupling of bound states to continuum induced by generic potential which is oscillating in time. (b) fiber with oscillating diameter (dispersion) which induces wavepackets that are localized in time thanks to a self-induced potential given by the nonlinearity to couple into the radiation continuum at characteristic resonant frequencies.
