# Supplementary material for: Parametric excitation of multiple resonant radiations from localized wavepackets
Source: Sci Rep. 2015 Mar 24;5:9433. doi: 10.1038/srep09433 (PMC4371105; doi:10.1038/srep09433)
Supplement: Supplementary Information — s [file srep09433-s1.pdf]

Parametric excitation of multiple resonant radiations from localized wavepackets: Supplementary information

Matteo Conforti[1,*], Stefano Trillo[2], Arnaud Mussot[1], and Alexandre Kudlinski[1]

[1]*PhLAM/IRCICA, CNRS-Université Lille 1,*

UMR 8523/USR 3380,

F-59655 Villeneuve d'Ascq, France

[2]*Dipartimento di Ingegneria,*

Università di Ferrara,

Via Saragat 1, 44122 Ferrara, Italy

email: matteo.conforti@univ-lille1.fr

I. DERIVATION OF THE RESONANCES

In order to derive the equation of the resonances [Eq. (1) of the paper], we start from the following conservative nonlinear Schrödinger equation (NLSE)

$$i\partial_z u + d(i\partial_t)u - \frac{\delta\beta_2(z)}{2}\partial_t^2 u + \gamma|u|^2 u = 0, \tag{1}$$

$$d(i\partial_t) = \sum_{n\geq 2}\frac{\beta_n}{n!}(i\partial_t)^n, \quad D(\omega) = \sum_{n\geq 2}\frac{\beta_n}{n!}\omega^n. \tag{2}$$

where t stands for time measured in the frame moving at natural group velocity of light $V_g = dk/d\omega^{-1}$ [1], γ is the nonlinear coefficient, $\beta_n = d^n k/d\omega^n$ is the n-th order average dispersion, $d(i\partial_t)$ and $D(\omega)$ are the average dispersion operators in time and frequency domain (Fourier transform), respectively. In Eq. (1) the average group-velocity dispersion (GVD) β_2 is perturbed by the periodic modulation $\delta\beta_2(z)$ and by higher-order dispersive effects (β_n, $n \geq 3$).

We consider a localized wave-packet (either a soliton or a shock front) that moves with characteristic velocity $V_s = 1/\Delta k_1$, or in other words a solution $u_s(z,t) = A(\tau)\exp(iqz)$ of the unperturbed Eq. (1) which has nonlinear wavenumber q and is stationary in the

reference frame $\tau = t - \Delta k_1 z$. In terms of this new variable Eq. (1) reads as:

$$i\partial_z u + \hat{d}(i\partial_\tau)u - \frac{\delta\beta_2(z)}{2}\partial_\tau^2 u + \gamma|u|^2 u = 0, \tag{3}$$

$$\hat{d}(i\partial_\tau) = \sum_{n\geq 2}\frac{\beta_n}{n!}(i\partial_\tau)^n - \Delta k_1 i\partial_\tau, \quad \hat{D}(\omega) = \sum_{n\geq 2}\frac{\beta_n}{n!}\omega^n - \Delta k_1\omega. \tag{4}$$

When the perturbation is effective, the solution can be decomposed into the localized wave and radiation modes $g(z,\tau)$

$$u(z,\tau) = A(\tau)e^{iqz} + g(z,\tau). \tag{5}$$

Assuming the radiation modes to be weak ($|g| \ll |A|$), the linearization of Eq. (1) around the localized wave yields

$$i\partial_z g + \hat{d}(i\partial_\tau)g - \frac{\delta\beta_2(z)}{2}\partial_\tau^2 g + \gamma\left(u_s^2 g^* + 2|u_s|^2 g\right) = \frac{\delta\beta_2(z)}{2}\partial_t^2 u_s - \hat{d}_H(i\partial_\tau)u_s, \tag{6}$$

where $\hat{d}_H(i\partial_\tau) = \sum_{n\geq 3}\frac{\beta_n}{n!}(i\partial_\tau)^n$ accounts for the perturbation due to higher-order dispersive terms. The RHS of Eq. (6) represents the driving force for the evolution of the radiation modes. Importantly, the effective wavenumber of this forcing is not simply the wavenumber of the localized wave-packet but is also affected by the quasi-momentum associated with the periodic modulation of the second order dispersion.

Let us focus first on the free evolution of the system, described by the LHS of Eq. (6). Without loss of generality, the radiation can be searched in the form

$$g(z,t) = \left[a(z)e^{i(kz-\omega\tau)} + b^*(z)e^{-i(kz-\omega\tau)}\right]\exp\left[iqz + i\frac{\omega^2}{2}\int_0^z \delta\beta_2(s)ds\right]. \tag{7}$$

We find the following system that rules the free evolution [i.e. neglecting the forcing term corresponding to RHS of Eq. (6)] of the dispersive waves

$$i\partial_z\begin{bmatrix} a \\ b\end{bmatrix} + \begin{bmatrix} \hat{D}(\omega) - k - q + 2\gamma|A|^2 & \gamma A^2\exp\left[-i\omega^2\int_0^z \delta\beta_2(s)ds\right] \\ -\gamma(A^*)^2\exp\left[i\omega^2\int_0^z\delta\beta_2(s)ds\right] & -\left(\hat{D}(-\omega) + k - q + 2\gamma|A|^2\right)\end{bmatrix}\begin{bmatrix} a \\ b\end{bmatrix} = 0. \tag{8}$$

The dispersion relation of the linear waves $k = k(\omega)$ can be found by setting the determinant of the matrix equal to zero. In terms of the odd and even dispersive contributions ($\hat{D}_o(\omega) = \hat{D}(\omega) - \hat{D}(-\omega)$ and $\hat{D}_e(\omega) = \hat{D}(\omega) + \hat{D}(-\omega)$, respectively), we find

$$k_\pm(\omega) = \frac{\hat{D}_o(\omega)}{2} \pm \frac{1}{2}\sqrt{\left[\hat{D}_e(\omega) - 2q + 2\gamma|A|^2\right]\left[\hat{D}_e(\omega) - 2q + 6\gamma|A|^2\right]}. \tag{9}$$

For the soliton, Eq. (9) holds true with $A = 0$ since radiation is not temporally overlapped with the soliton (it grows on soliton tails where A exponentially vanishes) and $q = \gamma P/2$ [1], being P the peak power of the soliton. Therefore we obtain

$$k(\omega) = \hat{D}(\omega) - \gamma \frac{P}{2}. \tag{10}$$

For the shock wave, the radiation modes are amplified out of noise close to the leading edge of the dispersive shock wave. Therefore they propagate over the flat-top background A_0 (power $P_b = |A_0|^2$), which develops as a result of the steepening of the pulse edges [2], which is in turn responsible for the shock formation. In this case we can set $q = \gamma P_b$, i.e. the wavenumber of the gray soliton associated to the leading edge of the dispersive shock wave. In this case Eq. (9) yields

$$k_{\pm}(\omega) = \frac{\hat{D}_o(\omega)}{2} \pm \frac{1}{2}\sqrt{\hat{D}_e(\omega)\left[\hat{D}_e(\omega) + 4\gamma|A_0|^2\right]}. \tag{11}$$

In the shock case the presence of the background generates two symmetric branches of the dispersion relation. This fact accounts for the four wave mixing between the dispersive waves and the pump. Usually the amplitude of the two symmetric waves (a and b) are orders of magnitude different, so that only one branch of dispersion relation (k_+ in our case) turns out to be relevant. Under the hypothesis $|A_0|^2 \ll |\hat{D}_e|$, we can expand in Eq. (11) the square root to obtain, for the relevant branch,

$$k(\omega) = \hat{D}(\omega) + \gamma P_b. \tag{12}$$

The forcing term F arising from the RHS of Eq. (6) which is effective for the growth of the radiation modes with complex amplitudes a and b, turns out to be

$$F = \frac{\delta\beta_2(z)}{2} \exp\left[-i\frac{\omega^2}{2}\int_0^z \delta\beta_2(s)ds\right] \partial_t^2 A - \exp\left[-i\frac{\omega^2}{2}\int_0^z \delta\beta_2(s)ds\right] \hat{d}_H(i\partial_\tau)A. \tag{13}$$

By considering a periodic $\delta\beta_2(z)$ with period Λ, we can expand the perturbation in Fourier series of the form $\delta\beta_2(z) = \sum_l c_l e^{il\frac{2\pi}{\Lambda}z}$, and consequently expand the exponential in Eq. (13) as

$$\exp\left[-i\frac{\omega^2}{2}\int_0^z \delta\beta_2(s)ds\right] = \sum_m d_m e^{im\frac{2\pi}{\Lambda}z}. \tag{14}$$

Therefore we cast Eq. (13) in the form

$$F = \partial_\tau^2 A \sum_{l,m} c_n d_m e^{i(l+m)\frac{2\pi}{\Lambda}z} - \hat{d}_H(i\partial_\tau)A \sum_m d_n e^{im\frac{2\pi}{\Lambda}z}, \tag{15}$$

which allow to recognize two different driving terms for the growth of radiation modes. The first one comes from the modulation of the GVD, whereas the second one from higher-order dispersive terms. Importantly, even in the case of sinusoidal modulation, the exponential term (14) generates an infinite set of Fourier harmonics.

Coupling of energy into the radiation modes (dispersive waves) efficiently occurs when their wavenumber $k(\omega)$ equals the wavenumber of the forcing term. This leads to the following resonance condition (quasi-phase-matching)

$$k(\omega) = \frac{2\pi}{\Lambda} m, \quad m = 0, \pm 1, \pm 2, \ldots \tag{16}$$

or, equivalently, in the form of Eq. (1) of the paper:

$$\hat{D}(\omega) - k_{nl} = \frac{2\pi}{\Lambda} m, \quad m = 0, \pm 1, \pm 2, \ldots \tag{17}$$

where $k_{nl} = \gamma P/2$ and $k_{nl} = -\gamma P_b$, for the soliton and the shock configuration, respectively.

Equation (16) retains its validity for any general dispersion profile. However, in our experiments the dispersive operator can be truncated to the first correction to GVD, i.e. third-order dispersion β_3, whereas all the higher-order dispersive terms can be safely neglected. In this case, Eq. (16) can be cast, for the soliton configuration, in the final form

$$\frac{\beta_3}{6}\omega^3 + \frac{\beta_2}{2}\omega^2 - \Delta k_1 \omega - \gamma \frac{P}{2} = \frac{2\pi}{\Lambda} m, \tag{18}$$

whereas for the shock configuration, we obtain

$$\frac{\beta_3}{6}\omega^3 + \frac{\beta_2}{2}\omega^2 - \Delta k_1 \omega + \gamma P_b = \frac{2\pi}{\Lambda} m. \tag{19}$$

II. PROPERTIES OF THE DISPERSION OSCILLATING FIBERS

Two different DOFs with different lengths and modulation periods have been fabricated for the experiments. The fiber used for the soliton experiment, labelled DOF#1 here, is 150 m long and has a modulation period $\Lambda = 5$ m. Its outer diameter, which longitudinal evolution is displayed in Fig. 5a of the manuscript, oscillates between 110 and 123 μm. The inset in Fig. 1a shows a scanning electron microscope (SEM) image of the DOF cross section. It has two bigger holes around the core allowing to increase its form birefringence and to ensure a polarization-maintaining behaviour. Figure 1a shows the full dispersion curve simulated with a commercial finite-element mode solver for the maximum and minimum

diameters (red and blue lines respectively), for the neutral axis excited in experiments. The black line represents the average dispersion over the whole DOF length. The average zero-dispersion wavelength is located at 1064.5 nm. Its attenuation was measured to be 0.75 dB at 1064 nm and its nonlinear parameter was calculated to be $\gamma = 10$ (W km)$^{-1}$ at this wavelength. The DOF used for the dispersive shock wave experiment, labelled DOF#2, is 50 m long and has a modulation period $\Lambda = 0.5$ m. Its outer diameter (Fig. 5d of the manuscript) oscillates between 110 and 122 μm. It is based on the same design than DOF#1 although the geometrical parameters of the cross-section are slightly different. Figure 1b shows the simulated dispersion curves for the maximum and minimum diameters (red and blue lines respectively) as well as the average one (black line). The average zero-dispersion wavelength is located at 1062.5 nm. The overall attenuation is 0.35 dB at 1064 nm, and its nonlinear parameter is $\gamma = 10$ (W km)$^{-1}$ at this wavelength.

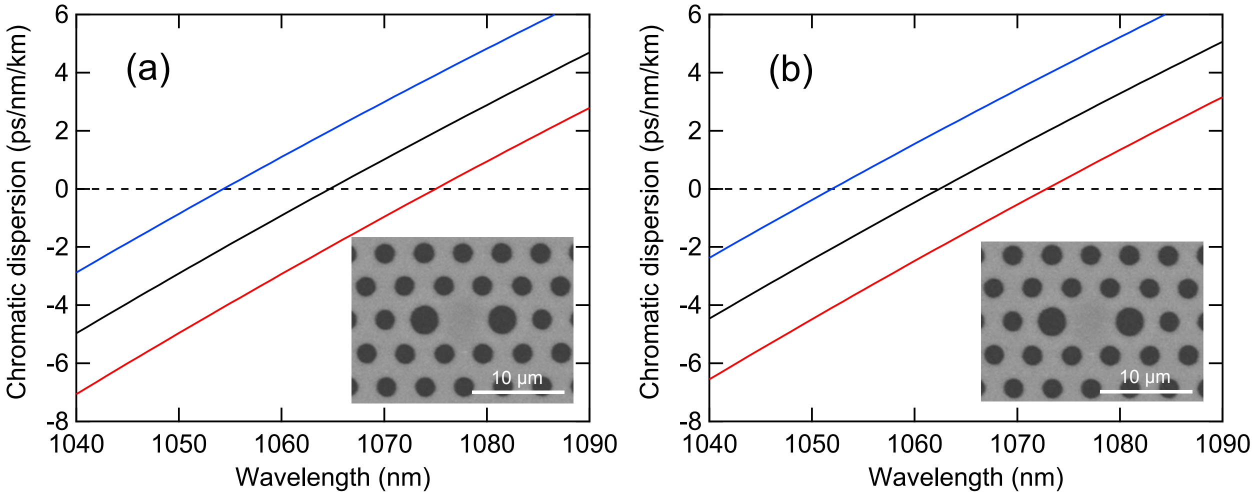

FIG. 1: Simulated dispersion curves of the DOFs used for the soliton (a, DOF#1) and dispersive shock wave (b, DOF#2) experiments. Red and blue lines correspond to the maximum and minimum diameters, respectively. Black lines correspond to the average dispersion curves over the whole DOF lengths. Insets show SEM images of the DOF cross sections at the maximum diameters.

Parameter	Soliton	Shock
β_2 (ps^2/km)	-1.2	2.9
$\delta\beta_2$ (ps^2/km)	1.2	1.2
β_3 (ps^3/km)	0.0716	0.0645
β_4 (ps^4/km)	$-1.1 \cdot 10^{-4}$	$-1.1 \cdot 10^{-4}$
γ (W^{-1}km^{-1})	10	10
α (dB/km)	5	7
Λ (m)	5	0.5
L (m)	150	50

TABLE I: Parameters of the fibers employed in the numerical simulations

III. NUMERICAL SIMULATIONS AND FIBER PARAMETERS

The parameters of the fibers that arise from fiber characterization and have been used in numerical simulations are listed in table 1. In the illustrating example reported in Figs. 2 and 3 of the manuscript, only the elements which are essential for the description of the basic phenomenon have been considered. In this case the simulations has been performed by integrating the nonlinear Schrödinger equation (NLSE) reported in the method section, i.e. the basic NLSE with additional perturbations due to third-order dispersion β_3 and the periodic GVD $\delta\beta_2(z)$. For simplicity, the initial conditions have the following hyperbolic secant shapes $E(t, z = 0) = \sqrt{P_0}\text{sech}\left(\frac{1.76t}{T_0}\right)$, with $T_0 = 150$ fs and $P_0 = 15$ W, for the soliton configuration, and $T_0 = 280$ and $P_0 = 100$ W for the shock wave configuration. The spectrograms [Fig. 3 in the paper] have been calculated by using a Gaussian pulse as a gate with duration of 1.6 ps.

When comparing directly with the experimental results [see Fig. 4c,f in the paper] we make use of the following extended NLSE [1], which accounts also additional effects such as higher order dispersion effects, Raman effect, self-steepening, and fiber losses

$$\frac{\partial E(z,t)}{\partial z} = -i\frac{\beta_2(z)}{2}\frac{\partial^2 E(z,t)}{\partial t^2} + \frac{\beta_3}{6}\frac{\partial^3 E(z,t)}{\partial t^3} + i\frac{\beta_4}{24}\frac{\partial^4 E(z,t)}{\partial t^4} - \frac{\alpha}{2}E(z,t) + i\gamma\left(1 + i\tau_s\frac{\partial}{\partial t}\right) \times \left(E(z,t)\int R(t')|E(z,t-t')|dt'\right), \quad (20)$$

with β_n the nth order dispersion terms, α the linear losses, $\tau_s = 1/\omega_p$ with ω_p the central pulsation of the pulse, $R(t)$ the full nonlinear response function that includes the instanta-

neous (Kerr) and delayed (Raman [3]) contributions with fractional weights $f_{Kerr} = 0.82$ and $f_{Raman} = 0.18$, respectively [1]. Here $\beta_2(z) = \beta_2 + \Delta\beta_2 \sin(2\pi z/\Lambda)$ and we checked through numerical simulations that all other parameters can be assumed to be constant along the fiber length. Indeed, the modulation of the nonlinear coefficient for instance is about 10 %, which is about one order of magnitude lower than the β_2 one [4].

In this case we also use initial conditions that accurately describe the pulses injected in the fiber, which has been experimentally characterized by means of the frequency resolved optical gating (FROG) system. In particular the best fit with FROG data gives slightly chirped input pulses of the following form: $E(t, z = 0) = \sqrt{P_0}\mathrm{sech}\left(\frac{1.76t}{T_0}\right)\exp(\frac{-iCt^2}{2T_0^2})$ for the soliton configuration with $T_0 = 150$ fs and $C = 0.35$, and $E(t, z = 0) = \sqrt{P_0}\exp\left(\frac{-1.665(1+iC)t^2}{2T_0^2}\right)$ for the shock configuration with $T_0 = 280$ fs and $C = 1.24$. The outcome of the numerical integration of Eq. (20) with such initial conditions are directly compared with the experimental results in Fig. 4c,f of the paper.

Using the realistic simulation parameters given above, we have also simulated the spectral output against pump peak power in both the soliton and dispersive shock wave configuration, respectively. The results, displayed in Fig. 2a,b are directly comparable with the experimental maps reported in Figs. 5b,e of the paper. In both cases the quantitative agreement with experimental data is excellent and confirms the progressive excitation of multiple resonances induced the periodic driving with increasing peak power.

Finally, we emphasize that we have compared the experimental results with the simulations of the full model [Eq. (20)] in order to have a better accuracy over all the details. However, we have verified that no significant difference arises when integrating the simpler NLSE reported in methods, since the additional terms (steepening, Raman effect, losses, fourth-order dispersion) are indeed negligible in our regime. In particular the parametric excitation of the resonances that we observe in the experiments and in the numerics are indeed quantitatively and accurately explained on the basis of this simpler NLSE.

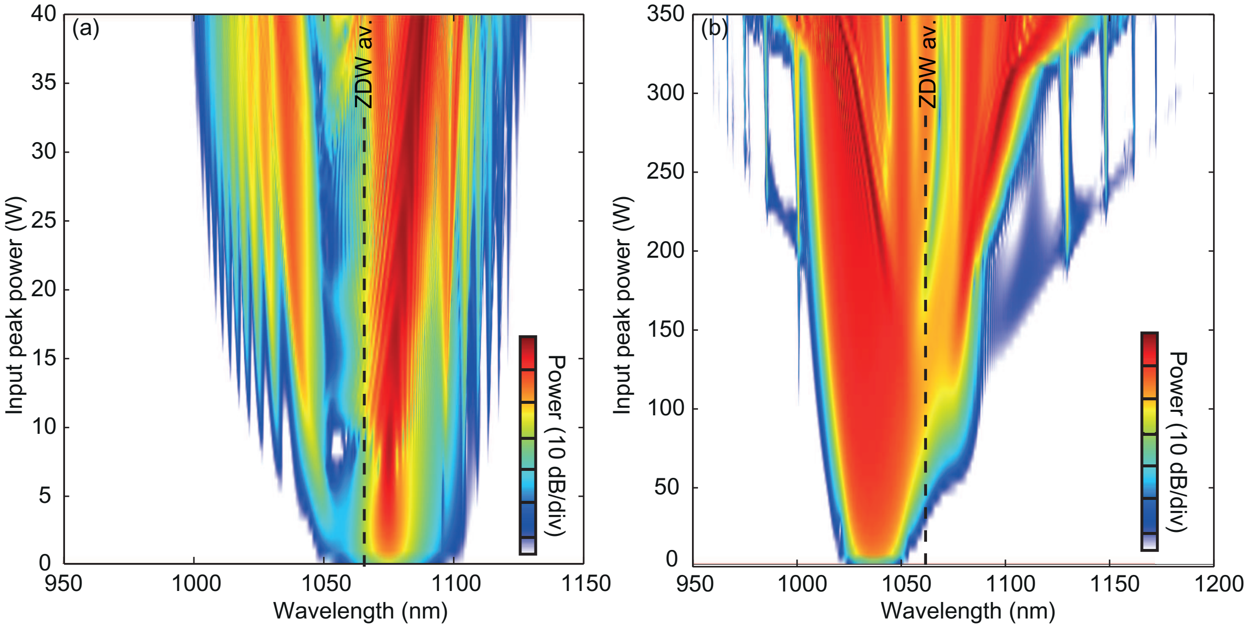

FIG. 2: Colormap of the output spectrum against the input power obtained by means of numerical integration of Eq. (20): a. soliton configuration; b. shock configuration. The dashed vertical lines indicates the average zero dispersion wavelength (ZDW). The maps in panels a and b are directly comparable with the experimental results in Figs. 5b and 5e of the paper, respectively.

[1] Agrawal, G. P. *Nonlinear Fiber Optics*, Academic Press (5th edition, 2012).

[2] Conforti, M., Baronio, F. & Trillo, S. Resonant radiation shed by dispersive shock wave, *Phys. Rev. A* **89**, 013807 (2014).

[3] Hollenbeck, D. & Cantrell, C. D. Multiple-vibrational-mode model for fiber-optic Raman gain spectrum and response function, *J. Opt. Soc. Am. B* **19**, 2886 (2002).

[4] Droques, M., Kudlinski, A., Bouwmans, G. , Martinelli, G. & Mussot, A. Experimental demonstration of modulation instability in an optical fiber with a periodic dispersion landscape, *Opt. Lett.* **37**, 4832 (2012).
